# Artemether/Lumefantrine for the Treatment of *P. malariae* in a Patient on Hemodialysis

**DOI:** 10.1155/2019/1326171

**Published:** 2019-02-12

**Authors:** Rita Patrocínio-Jesus, João Cunha, Diva Trigo, Bárbara Flor-de-Lima, Patrícia Pacheco

**Affiliations:** Infectious Diseases Department, Hospital Prof. Doutor Fernando Fonseca, EPE, IC 19, 2720-276 Amadora, Portugal

## Abstract

The combination of artemether/lumefantrine is indicated for the treatment of acute uncomplicated *Plasmodium falciparum* malaria. There have been no clinical trials to assess the efficacy of this medication in patients with renal impairment. While it is unlikely that artemether/lumefantrine would be removed during dialysis, clinical experience regarding drug use in this setting is limited. In this article, the authors report successful treatment of *Plasmodium malariae* malaria on a patient with end-stage kidney disease undergoing hemodialysis.

## 1. Introduction

The combination of artemether/lumefantrine is indicated for the treatment of acute uncomplicated *Plasmodium falciparum* malaria [1, 2]. No clinical trials have been conducted in patients with renal impairment, although no significant renal excretion of its components was reported in healthy volunteers [3]. Moreover, no data exist on these drugs dialyzability but physicochemical characteristics of artemether/lumefantrine suggest that significant drug removal is unlikely during dialysis [4]. Clinical experience is limited and, to our knowledge, few cases have been published reporting its use in patients with acute kidney disease [5].

Despite no data provided by the manufacturers about the effectiveness of artemether/lumefantrine against *Plasmodium malariae*, a study performed in Central Africa showed high rates of cure of nonfalciparum malaria with this combination [6] and current guidelines recommend the treatment of uncomplicated *P. malariae* malaria with an artemisinin-based combination therapy [1].

## 2. Case Presentation

We describe a clinical case of a 56-year-old woman from Angola, with diabetic end-stage kidney disease under maintenance hemodialysis and chronic anemia with frequent blood transfusion requirements, who presented with lumbar back pain and lower extremity muscle weakness for 3 months. The patient reported myalgia, rigors, and epigastric pain for 1 month, which had worsened in the previous 7 days, at which time she travelled to Portugal. At presentation, the patient's vital signs were normal, and physical examination was remarkable for pallor and proximal weakness of the lower limbs. Blood tests on admission revealed anemia (hemoglobin 6 g/dL, mean corpuscular volume 67.1 fL, mean corpuscular hemoglobin concentration 20.5 pg, hematocrit 19.6%, and red cell distribution width 28%), leukocyte count 8.6 × 10^9^/L and platelet count 77 × 10^9^/L, C-reactive protein 26.88 mg/dL, creatinine 8.36 mg/dL, and urea 141 mg/dL, with no evidence of hemolysis. The peripheral blood smear revealed trophozoites and schizonts of *Plasmodium malariae*, and the patient was treated with artemether/lumefantrine for 3 days, under cardiac monitoring with electrocardiogram and blood potassium monitoring. No infected erythrocytes were identified on peripheral blood smear after treatment conclusion, and no adverse events were reported.

## 3. Discussion

In conclusion, artemether/lumefantrine may be safe for patients with end-stage kidney failure. Nevertheless, studies on end-stage kidney disease patients are warranted to support its safe and efficacious use. Close monitoring of these patients with a multidisciplinary team is essential.
